# Intrathecal curcumin attenuates pain hypersensitivity and decreases spinal neuroinflammation in rat model of monoarthritis

**DOI:** 10.1038/srep10278

**Published:** 2015-05-19

**Authors:** Jun-Jie Chen, Lin Dai, Lin-Xia Zhao, Xiang Zhu, Su Cao, Yong-Jing Gao

**Affiliations:** 1 Department of Anesthesiology, Affiliated Hospital of Nantong University, Nantong, Jiangsu 226001, China; 2 Pain Research Laboratory, Institute of Nautical Medicine, Nantong University, Nantong, Jiangsu 226019, China; 3 Department of Rheumatology, Affiliated Hospital of Nantong University, Nantong, Jiangsu 226001, China

## Abstract

Curcumin is a major component of turmeric and reportedly has anti-inflammatory and anti-oxidant effects. Neuroinflammation has been recognized to play an important role in the pathogenesis of various diseases in the central nervous system. Here we investigated the anti-nociceptive and anti-neuroinflammatory effect of curcumin on arthritic pain in rats. We found that repeated oral treatment with curcumin, either before or after complete Freund’s adjuvant (CFA) injection, dose-dependently attenuated CFA-induced mechanical allodynia and thermal hyperalgesia, but had no effect on joint edema. Repeated intrathecal injection of curcumin reversed CFA-induced pain hypersensitivity. Furthermore, such a curcumin treatment reduced CFA-induced activation of glial cells and production of inflammatory mediators [interleukin-1β (IL-1β), monocyte chemoattractant protein-1 (MCP-1), and monocyte inflammatory protein-1 (MIP-1α)] in the spinal cord. Curcumin also decreased lipopolysaccharide-induced production of IL-1β, tumor necrosis factor-α, MCP-1, and MIP-1α in cultured astrocytes and microglia. Our results suggest that intrathecal curcumin attenuates arthritic pain by inhibiting glial activation and the production of inflammatory mediators in the spinal cord, suggesting a new application of curcumin for the treatment of arthritic pain.

Curcumin is a major bioactive component of turmeric and has been used as an oral and topical medication to treat a wide variety of ailments, including pain, rheumatoid arthritis, and cancer^1^,^2^. Emerging experimental evidence indicates that curcumin has analgesic effect on both acute inflammatory pain and chronic neuropathic pain. For example, acute systemic treatment with curcumin reduced formalin-induced defensive behaviors^3^ and acetic acid-induced visceral nociception^4^. Repeated systemic treatment with curcumin alleviated neuropathic pain induced by sciatic nerve injury^5^, streptozotocin^6^, and cisplatin^7^. Curcumin also reduced paw inflammation in collagen-induced arthritis^8^. Inflammation of the joint is one of the major causes of chronic pain^9^, and whether curcumin has analgesic effect on articular inflammatory pain and the underlying mechanisms remain unclear.

Curcumin has been demonstrated to have a variety of biological activities, including anti-inflammatory, anti-oxidant, and anti-tumor actions^10^,^11^. *In vitro* studies showed that curcumin inhibits the production of proinflammatory cytokines [e.g. interleukin-1β (IL-1β), tumor necrosis factor-α (TNF-α)], and chemokines [e.g. monocyte chemoattractant protein-1 (MCP-1), monocyte inflammatory protein-1 (MIP-1α)] stimulated by lipopolysaccharide (LPS) in astrocytes, monocytes, or alveolar macrophages^12^,^13^. Curcumin is also a potent inhibitor of the mitogen-activated protein kinases (MAPKs) and NF-κB^14^,^15^,^16^, which are critical in the transcriptional regulation of proinflammatory cytokine gene expression and are also important for the maintenance of chronic pain^17^,^18^.

Neuroinflammation has been demonstrated to play a pivotal role in the pathogenesis of chronic pain^19^,^20^,^21^. In response to peripheral inflammation, glial cells (astrocytes and microglia) are activated and produce multiple inflammatory mediators such as proinflammatory cytokines and chemokines^19^,^22^,^23^,^24^,^25^, which are involved in the regulation of synaptic transmission^26^,^27^,^28^. Inhibition of neuroinflammation that is mediated by glial cells attenuates inflammatory or neuropathic pain^19^,^20^,^21^. Whether curcumin can regulate the activity of glial cells and reduce the inflammation in the spinal cord in the setting of arthritic pain condition remains to be determined.

Complete Freund’s adjuvant (CFA) is frequently used to model arthritic disease, since it recapitulates many of the features of human rheumatoid arthritis^29^,^30^. In this study, we investigated the role of systemic or intrathecal treatment with curcumin on arthritic pain in the CFA-induced rat ankle joint monoarthritis (MA) model. We also explored the possible analgesic mechanisms of spinal injection of curcumin by assaying the activation of spinal glial cells and the production of inflammatory mediators both *in vivo* and *in vitro*.

## Results

### Systemic pre-treatment with curcumin attenuates CFA-induced mechanical allodynia and thermal hyperalgesia

**Figure 1:**
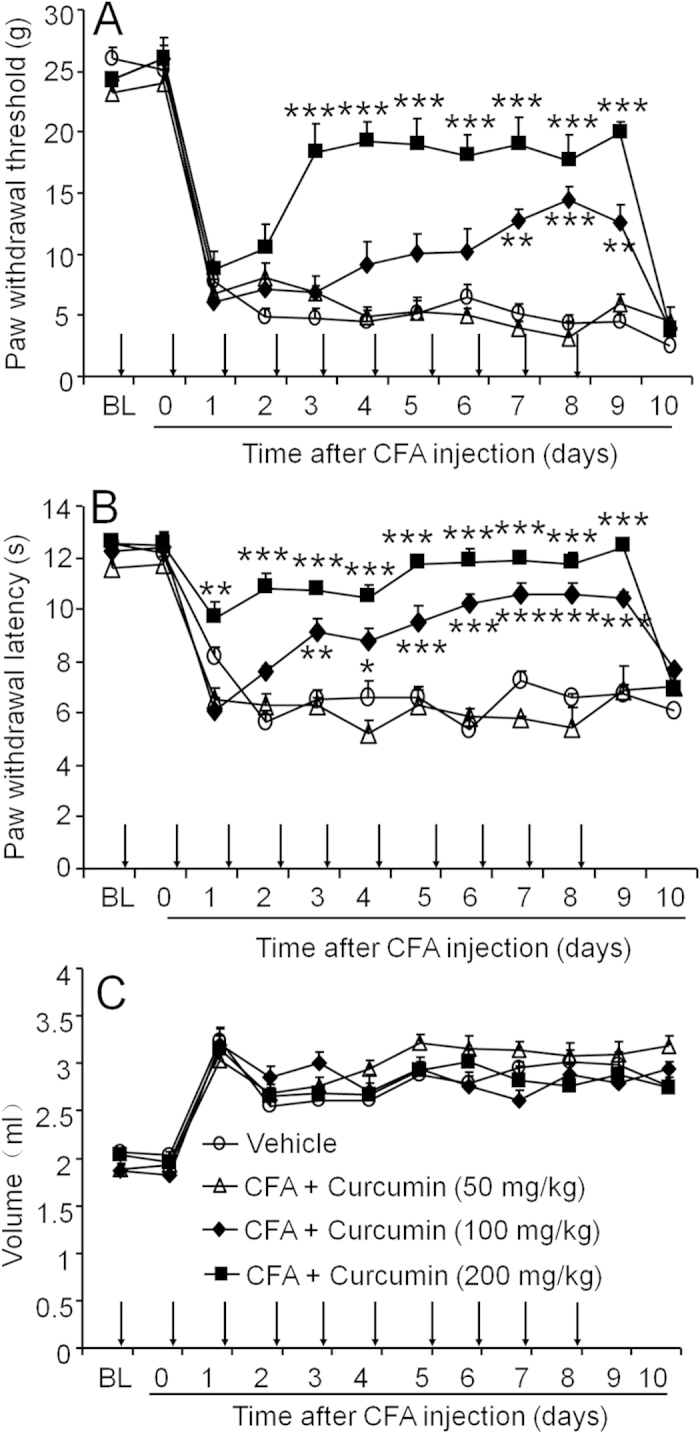
Systemic pre-treatment with curcumin attenuates CFA-induced mechanical allodynia and heat hyperalgesia. Rats were repeatedly injected with curcumin, started from 1 day before CFA injection, for 10 consecutive days. Curcumin dose-dependently attenuated mechanical allodynia (**A**) and heat hyperalgesia (**B**). However, CFA-induced paw edema was not significantly changed by curcumin (**C**). * P < 0.05, ** P < 0.01, *** P < 0.001, vs. vehicle, two-way ANOVA followed by Bonferroni test. n = 6 rats per group

As it has been reported previously^31^, intra-articular injection of CFA induced pain hypersensitivity and joint inflammation (Fig. 1), but injection of normal saline did not induce pain (data not shown). To examine the anti-nociceptive effect of curcumin, different doses of curcumin or vehicle was delivered by oral gavage daily for 10 consecutive days. Curcumin treatment was started 24 h before CFA injection. As shown in Fig. 1, repeated treatment with curcumin effectively and dose-dependently alleviated CFA-induced pain hypersensitivity. For mechanical allodynia (Fig. 1A), an analysis by two-way ANOVA revealed a significant effect of Treatment (F_3, 220_ = 44.50 and P < 0.0001), Time (F_11, 220_ = 114.9 and P < 0.0001), and Time × Treatment interaction (F_33, 220_ = 7.710 and P < 0.0001). The Bonferroni post hoc tests showed that curcumin at a dose of 200 mg/kg did not affect PWT in the first 2 days. However, an attenuation of mechanical allodynia appeared at 3 days and persisted until 9 days after CFA injection. Curcumin at 100 mg/kg reduced CFA-induced mechanical allodynia from 7 days to 9 days after CFA injection (Fig. 1A). Curcumin also attenuated CFA-induced thermal hyperalgesia (Fig. 1B, Treatment, F_3, 220_ = 106.2 and P < 0.0001; Time, F_11, 220_ = 57.23 and P < 0.0001; Interaction, F_33, 220_ = 7.739 and P < 0.0001). The Bonferroni post hoc tests showed that curcumin at a dose of 200 mg/kg reversed CFA-induced thermal hyperalgesia from 2 days to 9 days after CFA injection. Curcumin at the dose of 100 mg/kg also diminished CFA-induced heat hyperalgesia from 3 days to 9 days (Fig. 1B). The lowest dose of curcumin (50 mg/kg) did not significantly change mechanical allodynia or heat hyperalgesia at any time point (Fig. 1A,B). All the three doses of curcumin did not affect the ankle joint edema (Fig. 1C). The overall condition and body weight were comparable among all the groups (data not shown).

### Systemic post-treatment with curcumin reverses CFA-induced mechanical allodynia and thermal hyperalgesia

**Figure 2:**
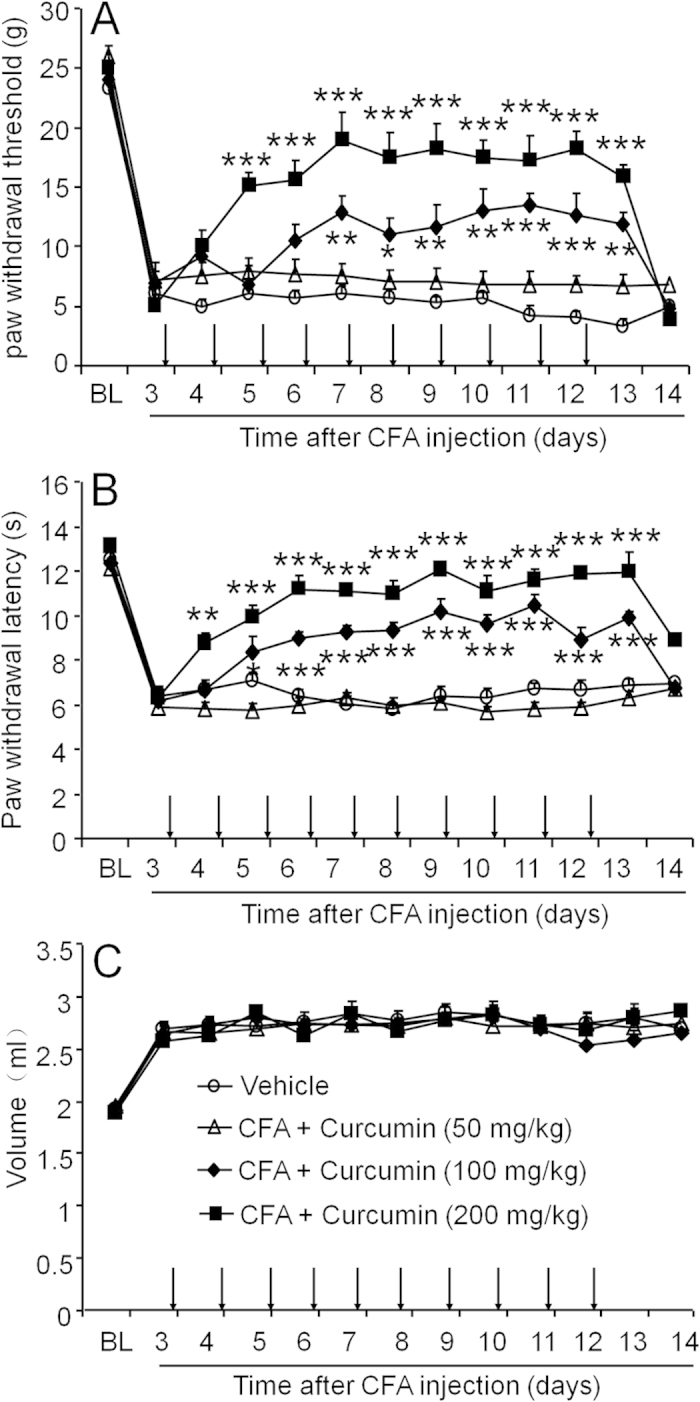
Systemic post-treatment with curcumin reverses CFA-induced mechanical allodynia and heat hyperalgesia. Rats were repeatedly injected with curcumin, started from 3 days after CFA injection, for 10 consecutive days. Curcumin dose-dependently reduced established mechanical allodynia (**A**) and heat hyperalgesia (**B**), but did not affect paw edema (**C**). ** P < 0.01, *** P < 0.001, vs. vehicle, two-way ANOVA followed by Bonferroni test. n = 6 rats per group.

We next evaluated the effect of curcumin on the alleviation of pain in established arthritis. We began curcumin treatment at 3 days after CFA injection when mechanical allodynia and thermal hyperalgesia were already fully developed and continued to treat daily for 10 consecutive days. Curcumin had a significant effect on mechanical allodynia (Fig. 2A, Treatment, F_3, 240_ = 50.28 and P < 0.0001; Time, F_12, 240_ = 62.95 and P < 0.0001; Interaction, F_36, 240_ = 5.764 and P < 0.0001). Furthermore, curcumin at a dose of 200 mg/kg attenuated mechanical allodynia from 5 days to 13 days after CFA injection. Curcumin at the dose of 100 mg/kg attenuated mechanical allodynia from 7 days to 13 days (Fig. 2A). For heat hyperalgesia (Fig. 2B), two-way ANOVA also showed significantly effects of treatment (F_3, 240_ = 121.0 and P < 0.0001), time (F_12, 240_ = 61.18 and P < 0.0001) and interaction (F_36, 240_ = 7.707 and P < 0.0001). The Bonferroni post hoc tests showed that curcumin (200 mg/kg) attenuated heat hyperalgesia from 4 days to 13 days after CFA injection (Fig. 2B). Treatment with curcumin did not change the ankle joint edema (Fig. 2C).

### Intrathecal post-treatment with curcumin reverses CFA-induced mechanical allodynia and heat hyperalgesia

**Figure 3:**
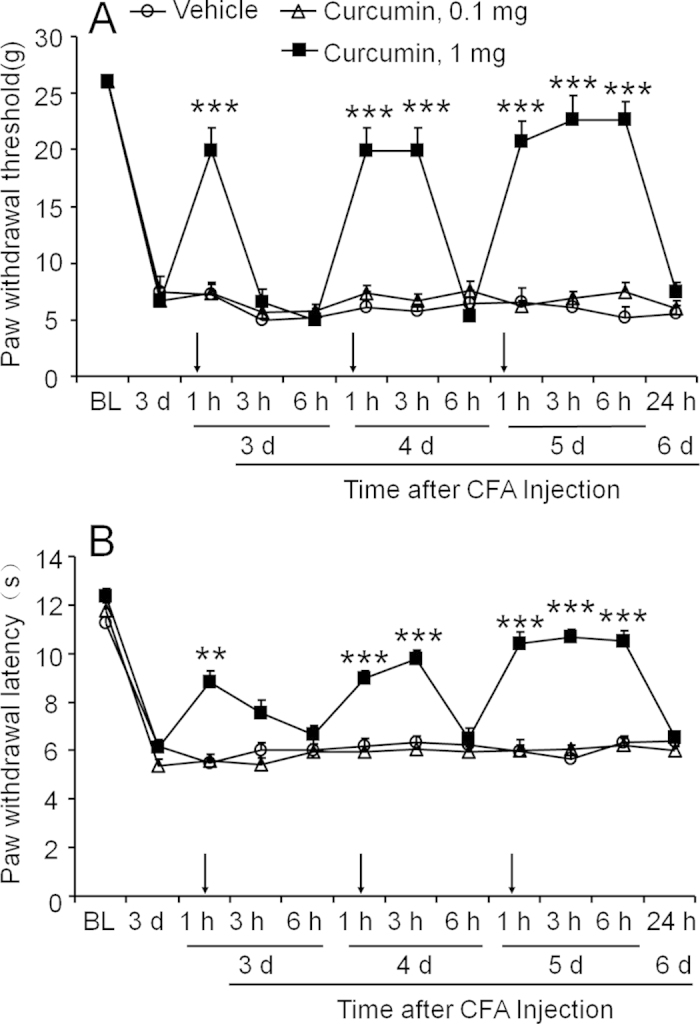
Intrathecal injection of curcumin reverses CFA-induced mechanical allodynia and heat hyperalgesia. Rats were repeatedly injected with curcumin intrathecally, started from 3 days after CFA injection, for 3 consecutive days. Curcumin at the dose of 1 mg reversed mechanical allodynia (**A**) and diminished heat hyperalgesia (**B**), whereas low dose (0.1 mg) did not show any effect. ** P < 0.01, *** P < 0.001, vs. vehicle, two-way ANOVA followed by Bonferroni test. n = 6 rats per group.

To investigate possible mechanisms of curcumin in the spinal cord, we delivered it by intrathecal injection. Curcumin was given daily for 3 consecutive days, starting from 3 days after CFA. Pain behavior was assessed at 1 h, 3 h, and 6 h after each injection. Curcumin showed a significant effect on mechanical allodynia (Fig. 3A, Treatment, F_2, 165_ = 122.8 and P < 0.0001; Time, F_11, 165_ = 80.56 and P < 0.0001; Interaction, F_22, 165_ = 16.7 and P < 0.0001) and heat hyperalgesia (Fig. 3B, Treatment, F_2, 143_ = 211.1 and P < 0.0001; Time, F_11, 143_ = 63.21 and P < 0.0001; Interaction, F_22, 143_ = 9.696 and P < 0.0001). The Bonferroni post hoc tests showed that the first injection of curcumin at a dose of 1 mg transiently reduced mechanical allodynia and heat hyperalgesia with the effect appearing at 1 h and vanished by 3 h. The second injection reversed mechanical allodynia and heat hyperalgesia from 1 h to 3 h, whereas the third injection reversed mechanical allodynia and heat hyperalgesia for more than 6 h, indicating that the effect of curcumin over time is cumulative. Curcumin at 0.1 mg had no effect on either mechanical or thermal sensitivity of MA rats (Fig. 3A,B).

### MA induces activation of glial cells and upregulation of proinflammatory cytokines and chemokines in the spinal cord

**Figure 4:**
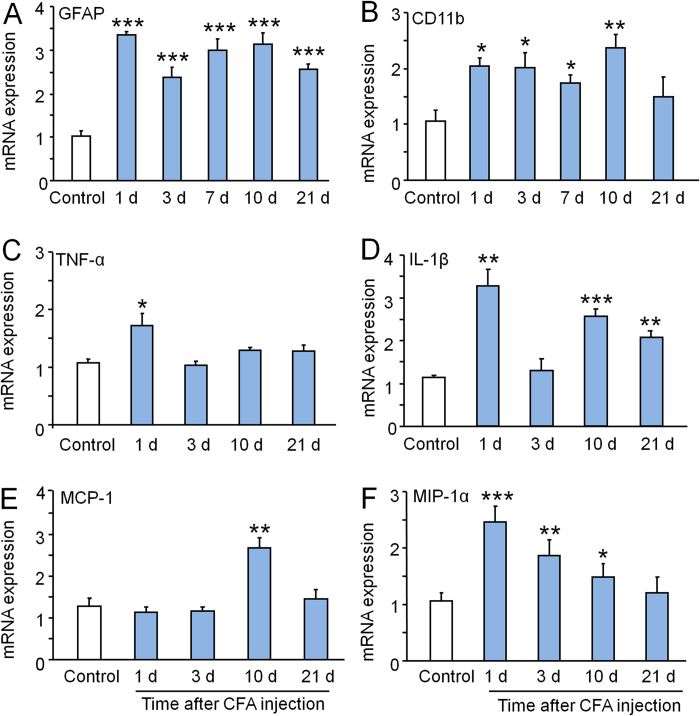
MA induces glial activation and upregulation of proinflammatory cytokines and chemokines in the spinal cord. MA increased the mRNA expression of the astrocytic marker GFAP (**A**), the microglial marker CD11b (**B**), TNF-α (**C**), IL-1β (**D**), MCP-1 (**E**), and MIP-1α (**F**) in the spinal cord. * P < 0.05, ** P < 0.01, *** P < 0.001, vs. control. One-way ANOVA followed by Bonferroni post hoc test. n = 5 rats per group.

It is increasingly recognized that non-neuronal cells such as glial cells play a critical role in the pathogenesis of chronic pain^32^,^33^. To investigate whether glial activation is involved in CFA-induced inflammatory pain, we checked the mRNA expression of the astrocytic marker GFAP, and the microglial marker CD11b in the spinal cord at different time points after CFA injection. As shown in Fig. 4, the expression of GFAP was increased from 1 day and maintained for more than 21 days after CFA (P < 0.001, one-way ANOVA, Fig. 4A); the expression of CD11b was also increased from 1 day, but maintained for 10 days after CFA injection (P < 0.001, one-way ANOVA, Fig. 4B).

Previous evidence has demonstrated that glial cells release a variety of mediators including proinflammatory cytokines and chemokines that contribute to the pathogenesis of pain^34^,^35^,^36^. We checked the mRNA expression of TNF-α, IL-1β, MCP-1, and MIP-1α. The mRNA of TNF-α was increased only at 1 day after CFA injection (P < 0.05, vs. control, Fig. 4C). IL-1β mRNA was increased at 1 day, 10 days, and 21 days after CFA injection (P < 0.01 or P < 0.001, vs. control, Fig. 4D). The chemokine MCP-1 was increased at 10 days after CFA injection (P < 0.01, vs. control, Fig. 4E). The expression of MIP-1α was increased at 1 day, 3 days and 10 days after CFA injection (P < 0.05 or P < 0.01, or P < 0.001, vs. control, Fig. 4F). These results suggest that CFA-induced inflammatory pain is associated with activation of glial cells (astrocytes and microglia) and the upregulation of proinflammatory cytokines and chemokines in the spinal cord.

### Intrathecal curcumin decreases CFA-induced activation of glial cells and upregulation of proinflammatory cytokines and chemokines in the spinal cord

**Figure 5:**
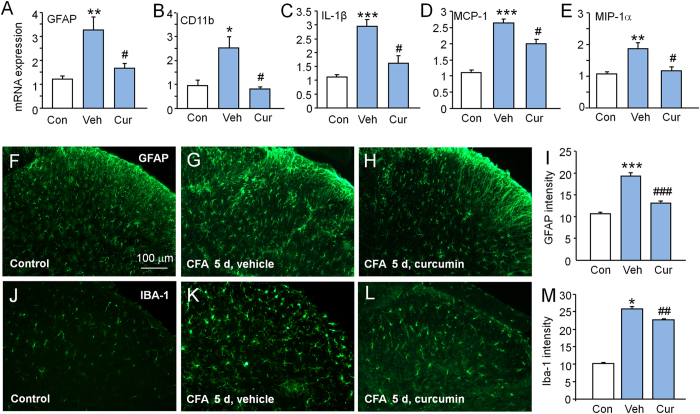
Intrathecal curcumin decreases glial activation and expression of inflammatory mediators in the spinal cord. Curcumin treatment decreased CFA-induced mRNA upregulation of GFAP (**A**), CD11b (**B**), IL-1β (**C**), MCP-1 (**D**), and MIP-1α (**E**) in the spinal cord. (F-H) Immunostaining showed that astrocytes appeared to be in a resting state in control animals (**F**), appeared larger and had more processes in vehicle-treated animals (**G**), and recovered in curcumin-treated animals (**H**). (**I**) The GFAP immunofluorescence intensity was decreased by curcumin (Cur), compared to vehicle (Veh). (**J**-**L**) Immunostaining showed that microglia appeared to be in a resting state in control animals (**J**), appeared larger and had more processes in vehicle-treated animals (**K**), and recovered in curcumin-treated animals (**L**). (**M**) The IBA-1 immunofluorescence intensity was decreased by curcumin. * P < 0.05, *** P < 0.001, compared with control. ## P < 0.01, ### P < 0.001, compared with vehicle. One-way ANOVA followed by Bonferroni post hoc test. n = 5 rats per group.

To investigate whether the anti-nociceptive effect of curcumin is associated with suppression of glial activation and downregulation of inflammatory mediators in the spinal cord, we checked GFAP, CD11b, IL-1β, MCP-1 and MIP-1α expression in the spinal cord at 6 h after the last injection at day 5. RT-PCR results showed that, compared with control animals, GFAP mRNA (Fig. 5A) and CD11b mRNA (Fig. 5B) were significantly increased in animals injected with vehicle. Curcumin treatment decreased GFAP mRNA expression (P < 0.05, vs. vehicle, Fig. 5A) and CD11b mRNA expression in the spinal cord (P < 0.05, vs. vehicle, Fig. 5B). Curcumin also significantly decreased IL-1β (P < 0.05, vs. vehicle, Fig. 5C), MCP-1 (P < 0.05, vs. vehicle, Fig. 5D), and MIP-1α (P < 0.05, vs. vehicle, Fig. 5E) mRNA levels in the spinal cord of MA rats.

We further examined the expression of GFAP and IBA-1 (another microglial marker) expression by immunofluorescence (IF) staining. In control animals, a few GFAP-positive astrocytes and IBA-1-positive microglia were seen (Fig. 5F,J). In the vehicle group, GFAP-IF was significantly increased and a large number of GFAP-positive astrocytes exhibited intense immunoreactivity and appeared hypertrophic with thick processes (Fig. 5G). Curcumin treatment decreased CFA-induced GFAP-IF upregulation in the spinal cord (P < 0.001, vs. vehicle, Fig. 5H,I). Similarly, IBA-1-IF was markedly increased in the vehicle group and the microglial processes were shortened and thickened (Fig. 5K). The CFA-induced activation of microglia was markedly reduced in spinal cord dorsal horn after curcumin treatment (P < 0.01, vs. vehicle, Fig. 5L,M). These results suggest that intrathecal curcumin attenuates activation of glia and expression of inflammatory mediators in the spinal cord in MA rats.

### Curcumin decreases production of inflammatory mediators in primary cultured astrocytes and microglia

**Figure 6:**
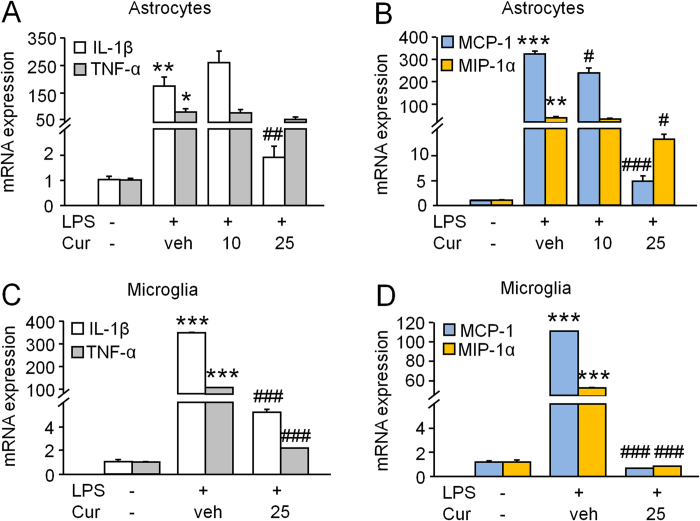
Curcumin decreases lipopolysaccharide (LPS)-induced upregulation of inflammatory mediators in cultured astrocytes and microglia. Cells were treated with different doses of curcumin or vehicle for 30 min, followed by incubation with LPS (1 μg/ml) for 3 h. Curcumin at the dose of 25 μM decreased LPS-induced upregulation of IL-1β (**A**), MCP-1 (**B**) and MIP-1α (**B**) in astrocytes. Curcumin at the dose of 25 μM also decreased production of proinflammatory cytokines (**C**) and chemokines (**D**) in microglia. * P < 0.05, ** P < 0.01, *** P < 0.001, compared to control. # P < 0.05, ## P < 0.01, ### P < 0.001, compared to vehicle. n = 3 for each treatment. Cur, curcumin; veh, vehicle.

To further verify the effect of curcumin on the expression of inflammatory mediators (IL-1β, TNF-α, MCP-1, and MIP-1α) in glial cells, we employed primarily cultures of astrocytes and microglia. To mimic the neuroinflammation *in vitro*, we incubated the cells with LPS (1 μg/ml). As shown in Fig. 6, the cytokines (Fig. 6A) and chemokines (Fig. 6B) were constitutively expressed in astrocytes. LPS incubation for 3 h dramatically increased the expression of these cytokines and chemokines. Pretreatment with curcumin at the dose of 10 μM for 30 min only decreased MCP-1 mRNA expression (Fig. 6B). The dose at 25 μM reduced LPS-induced mRNA upregulation of IL-1β (P < 0.01, vs. vehicle, Fig. 6A), MCP-1 (P < 0.001, vs. vehicle, Fig. 6B), and MIP-1α (P < 0.05, vs. vehicle, Fig. 6B), whereas TNF-α expression was not affected (Fig. 6A).

We then examined the effect of curcumin on the production of inflammatory mediators in microglia. LPS dramatically increased IL-1β and TNF-α expression by more than 100-fold (Fig. 6C). LPS also significantly increased the mRNA expression of MCP-1 and MIP-1α by more than 60-fold (Fig. 6D). Pretreatment with curcumin (25 μM) dramatically decreased the upregulation of IL-1β, TNF-α, MCP-1 and MIP-1α (Fig. 6C,D).

## Discussion

In this study, we investigated the anti-nociceptive and anti-neuroinflammatory effect of curcumin on CFA-induced pain hypersensitivity. Our results demonstrated that repeated treatment with curcumin orally, starting either before or after CFA injection, attenuated CFA-induced pain hypersensitivity in a dose-dependent manner. Furthermore, intrathecal injection of curcumin attenuated CFA-induced pain hypersensitivity and decreased the activation of spinal glial cells (astrocytes and microglia). The same treatment of curcumin also reduced CFA-induced production of proinflammatory cytokine (IL-1β) and chemokines (MCP-1 and MIP-1α) in the spinal cord. *In vitro* studies further showed the inhibitory effects of curcumin on the expression of inflammatory mediators in cultured astrocytes and microglia. Taken together, our data suggest that intrathecal curcumin may attenuate chronic inflammatory pain potentially via inhibiting the activation of glial cells and production of proinflammatory cytokines and chemokines in the spinal cord.

Previous studies have demonstrated that systemic administration of curcumin attenuated inflammatory pain^3^,^37^ and neuropathic pain^5^,^6^,^7^. Although a single treatment of curcumin alleviated acute pain induced by formalin or acidic acid^3^,^4^, only repetitive treatment of curcumin attenuated chronic pain^38^,^39^,^40^. In agreement with these reports, our current study showed that both pre-treatment and post-treatment with curcumin attenuated CFA-induced mechanical allodynia and heat hyperalgesia. Moreover, curcumin had a greater better effect on heat hyperalgesia. However, repetitive treatment with curcumin had no effect on joint edema, indicating that the analgesic effect of systemic curcumin is mainly mediated through a central effect.

Indeed, previous studies showed that curcumin is well absorbed, has good tissue penetration, and readily crosses the blood–brain-barrier^41^. Systemic administration of curcumin was associated with the suppression of brain nitrite^6^, spinal COX-2^39^, TNF-α and TNF receptor 1^42^ in rats with neuropathic pain, supporting that systemic curcumin may have central effect. In this study, our data showed that the first single intrathecal injection of curcumin transiently alleviated CFA-induced pain hypersensitivity, whereas repetitive injection showed prolonged analgesic effect. Intrathecal administration of curcumin also decreased formalin-induced flinching responses^43^. The findings provide direct evidence for spinal effect of curcumin, and therefore raise the possibility of curcumin as a novel analgesic for spinal delivery.

Non-neuronal cells such as immune cells and glial cells have been implicated in the pathogenesis of chronic pain^32^,^33^. Both astrocytes and microglia were activated in the spinal cord following peripheral nerve injury or tissue inflammation. The activated glial cells contribute to the enhancement and maintenance of chronic pain by releasing neuromodulators, such as growth factors, proinflammatory cytokines and chemokines^25^,^44^. Behavioral studies showed that blockade of the activation of spinal cord microglia and astrocytes prevents or delays the development of pain hypersensitivity^45^,^46^,^47^. In this study, we found that the astrocytic marker GFAP mRNA was rapidly (1 d) and persistently (>21 d) increased in the spinal cord after MA, whereas the microglial marker CD11b mRNA was increased from 1 day to 10 days, but not at 21 days. Immunostaining further support the activation of microglia and astrocytes in the spinal cord at 5 days after MA. In agreement with our results, Sun *et al* showed that astrocytes and microglia were activated in the spinal cord at 3 days after CFA-induced MA^48^. Interestingly, intrathecal curcumin inhibited astrocytic and microglial activation in the spinal cord. A recent study showed that systemic curcumin inhibited the activation of astrocytes in the spinal dorsal horn in neuropathic pain^40^. Additionally, curcumin also showed a significant reduction of GFAP in brain in an animal model for Alzheimer’s disease^49^ and a reduction of CD11b in injured brain tissue^50^. These data suggest that curcumin is involved in the regulation of glial function in the central nervous system.

Previous results demonstrated that curcumin inhibits LPS-induced MCP-1 expression in astrocytoma cells^12^, osteoblastic cells^51^, blood monocytes and alveolar macrophage^13^. Curcumin also reduced the release of various inflammatory mediators (IL-1β, IL-6, TNF-α, and MCP-1) in transwell co-culture of neurons and microglia^50^. Here we found that a high dose of curcumin significantly decreased the expression of IL-1β, TNF-α, MCP-1, and MIP-1α in both primary astrocytes and primary microglia, suggesting the anti-inflammatory effect of cucurmin on glial cells.

Evidence suggests that proinflammatory cytokines and chemokines are mainly produced by glial cells in the spinal cord. For example, TNF-α is dominantly expressed in microglia^52^, whereas MCP-1 and IL-1β are mainly expressed in astrocytes in the spinal cord^26^,^53^. Our *in vivo* data showed that CFA induced upregulation of several cytokines and chemokines in the spinal cord, with different time courses. Notably, TNF-α and MCP-1 were increased at 1 day or 10 days after MA, respectively, whereas the increase for MIP-1α and IL-1β persisted for more than 10 days or 21 days, respectively, suggesting that they may have distinct roles in different phases of inflammatory pain. Intrathecal injection of curcumin markedly inhibited the mRNA upregulation of IL-1β, MCP-1, and MIP-1α in the spinal cord at 5 days. Consistent with our results, curcumin decreased spinal TNF-α and TNFR1 protein in diabetic neuropathic pain^42^. It also reduced the expression of IL-1β, IL-6, TNF-α, and MCP-1 in brain after experimental traumatic brain injury^50^. These data support the inhibitory role of curcumin on the production of inflammatory mediators in the central nervous system.

It has been demonstrated that inhibition of TNF-α, IL-1β, MCP-1, or MIP-1α decreased pain hypersensitivity in neuropathic pain and inflammatory pain^26^,^54^,^55^,^56^. Several inflammatory mediators were shown to be involved in central sensitization. For example, TNF-α and MCP-1 enhance excitatory synaptic transmission, and IL-1β enhances excitatory synaptic transmission and decreases inhibitory synaptic transmission in dorsal horn neurons^26^,^28^, suggesting that they directly regulate neuronal activity. TNF-α and IL-1β can also further activate spinal astrocytes through the receptors TNFR1 and IL-1R, respectively^57^. Besides the inhibition of glial activation and production of inflammatory mediators, curcumin may also exert an anti-nociceptive effect through modulatory effects on brainstem adrenergic and serotonergic systems^38^, on spinal antioxidant enzymes^58^, or on transient receptor potential vanilloid 1 channel function in nociceptive neurons^37^.

In conclusion, our present study demonstrated that repetitive treatment with curcumin (orally or intrathecally) significantly attenuated CFA-induced pain hypersensitivity. Especially, intrathecal injection of curcumin showed more rapid and potent analgesic effect than systemic injection. Furthermore, our *in vivo* and *in vitro* data indicate that the analgesic effect of intrathecal curcumin may be produced by inhibition of the activation of astrocytes and microglia and the corresponding production of glial-derived inflammatory mediators in the spinal cord. Collectively, our results provide a novel implication of anti-nociceptive mechanism of curcumin.

## Methods

### Animals

Male Sprague-Dawley rats (200–250 g, Experimental Animal Center, Nantong University, China) were used for experiments. These rats were housed under controlled conditions with a 12:12 h light/dark cycle with food and water available ad libitum. Animal experiments were all conducted according to protocols approved by Animal Care and Use Committee of Nantong University. Prior to experimental manipulation, rats were allowed to acclimate to the housing facilities for at least 3 days.

### Induction of monoarthritis (MA)

MA was induced by an injection of CFA (Sigma-Aldrich, St. Louis, MO) into the unilateral ankle articular cavity^48^. For the injection, rats were anesthetized with isoflurane (RWD Life Science, Shenzhen, China). The right leg of the rat was held and the fossa of the lateral malleolus of the fibula was located. A 28 gauge needle was inserted vertically to penetrate the skin, and insert into the articular cavity from the gap between the tibiofibular and tarsus bone until a distinct loss of resistance was felt. CFA (50 μl) was injected into the joint. The volume of right ankle joint and the hindpaw was measured using the plethysmometer (Ugo Basile, Italy).

### Drug treatments

For oral administration (via gavage, p.o.), the treatment with curcumin (Sigma-Aldrich) began 1 day before or 3 days after CFA injection. Curcumin was dissolved in peanut oil and administrated daily for 10 days with a volume of 10 ml/kg each time. The oral doses of 50, 100, 200 mg/kg were chosen based on a previous report^3^. For intrathecal injection, the treatment with curcumin began 3 days after CFA injection. Curcumin was dissolved in dimethyl sulfoxide (DMSO) and administered intrathecally in a 20 μl solution volume and continued daily for 3 days. Intrathecal injections were performed as described previously^59^. In brief, animals were anesthetized with isoflurane, and the injection was made with a 30-gauge needle in the L4 and L5 intervertebral space to deliver the drug to the cerebral spinal fluid. Immediately after the needle entry into subarachnoid space, a brisk tail flick could be observed.

### Behavioral Analysis

Mechanical allodynia was determined using a series of calibrated von Frey hairs (Stoelting, Wood Dale, IL) and expressed as hindpaw withdrawal threshold (PWT). Rats were placed in boxes on an elevated metal mesh floor and allowed 30 min for habituation before examination. Von Frey hairs were applied to the central region of the plantar surface of one hindpaw in ascending order (1.4, 2, 4, 6, 8, 10, 15, and 26 g). The 50% paw withdrawal threshold was determined using Dixon’s up-down method^60^. Heat hyperalgesia was assessed by measuring paw withdrawal latency (PWL) in response to a radiant heat source. Animals were put in plastic boxes and allowed 30 min for habituation. PWL was tested using Hargreaves apparatus (IITC Life Science Inc., Woodland Hills, CA). The radiant heat intensity was adjusted so that basal PWL is between 10 and 14 s, with a cutoff of 20 s to prevent tissue damage.

### Cell culture and treatment

Primary microglial and astrocytes cultures were prepared from cerebral cortexes of neonatal rats (postnatal day 1, P1)^61^. The cerebral hemispheres were isolated and transferred to ice-cold Hank’s buffer, and the meninges were carefully removed. Tissues were then minced into 1 mm pieces, triturated, filtered through a 100 μm nylon screen, and collected by centrifugation at 3000 g for 5 min. For astrocytes culture, the cell pellets were resuspended in a medium containing 10% fetal bovine serum (FBS) in low-glucose Dulbecco’s Modified Eagle’s Medium (DMEM). After filtration through a 10 μm screen, the cells were plated into six-well plates at a density of 2.5 × 10^5^ cells/cm^2^, and cultured for 10–12 d. Once the cells were grown to 95% confluence, 0.15 mM dibutyryl cAMP (Sigma) was added to induce differentiation. The cells can be used 3 days later. For microglial culture, the cell pellets were dispersed with a pipette and resuspended in a medium containing 10% FBS in high-glucose DMEM. After trituration, the cells were filtered through a 10 μm screen, plated into 75 cm^2^ flasks. After 12–14 days, the flasks were shaken on a rotary shaker at 220 rpm for 4 h. The resulting cell suspension, rich in microglia, was placed in culture dishes in which the cells adhered after 30 min at 37 °C.

When the cells were ready, they were incubated with lipopolysaccharide (LPS) for 3 h. The treatment of the curcumin (10 μM and 25 μM) 12 was started 30 min prior to LPS treatment. After the treatments, the cells were collected for real-time PCR.

### Real-time quantitative PCR

Animals were rapidly killed after deep anesthesia with isoflurane. The L_4-5_ spinal segments were quickly removed and directly homogenized in Trizol reagent (Invitrogen, Carlsbad, CA). One microgram of total RNA was reverse transcribed using a mixture of random primers according to the manufacturer’s protocol (TaKaRa, Japan). The cDNA was amplified using the following primers: GFAP (forward): 5′-GAC CGC TTT GCT AGC TAC ATC G-3′ and (reverse): 5′-GGT TTC ATC TTG GAG CTT CTG C-3′; CD11b (forward): 5′-AGA GTG TGA TCC AGC TTG GTG AAA-3′ and (reverse): 5′-AGT TTT TGT CCT TCC ATT CAG-3′; TNF-α (forward): 5′–GGG TGA TCG GTC CCA ACA-3′ and (reverse): 5′-TGG GCT ACG GGC TTG TCA-3′; IL-1β (forward): 5′-CAA AAA TGC CTC GTG CTG TCT-3′ and (reverse): 5′-TGT ACA AAG CTC ATG GAG AAT ACC A-3′; MCP-1 (forward): 5′–TGC TGC TAC TCA TTC ACT GGC-3′ and (reverse): 5′-CCT TAT TGG GGT CAG CAC AG-3′; MIP-1α (forward): 5′-CCA CTG CCC TTG CTG TTC TT-3′ and (reverse): 5′- GCA AAG GCT GCT GGT TTC AA-3′; GAPDH (forward): 5′-TCC TAC CCC CAA TGT ATC CG-3′ and (reverse): 5′-CCT TTA GTG GGC CCT CGG-3′. The SYBR Premix Ex Taq™II kit (Takara) was used for all PCR reactions, which were run on a Rotor-Gene 6000 RT-PCR machine (Hamburg, Germany). The PCR amplifications were performed at 95 °C for 30 s, followed by 40 cycles at 95 °C for 5 s, 56 °C for 30 s, and 72 °C for 30 s. The melting curves were performed to validate the utility and specificity of each PCR product. Quantification was performed by normalizing Ct (cycle threshold) values with GAPDH Ct and analyzed with the 2^−ΔΔCT^ method.

### Immunohistochemistry

After defined survival times, rats were anesthetized by Chloral Hydrate (300 mg/kg, i.p.) and perfused through the ascending aorta with saline followed by 4% paraformaldehyde in 0.1 M phosphate buffer (PB, pH 7.4). The L_4–5_ segments of spinal cord were then removed, post-fixed in the same fixative overnight at 4 °C, and immersed from 10% to 30% gradient sucrose in PB for 24–48 h at 4 °C for cryoprotection. Spinal cord sections (30 μm, free floating) were cut in a cryostat and processed for immunofluorescence as we described previously^26^. The sections were first blocked with 2% goat serum for 2 h at room temperature, then incubated overnight at 4 °C with the following primary antibodies: GFAP antibody (mouse, 1:6,000; Millipore, Billerica, MA), IBA-1 antibody (rabbit, 1:3000, Wako, Tokyo, Japan). Following three 15 min rinses in 0.01 M PBS, The sections were then incubated for 2 h at room temperature with Cy3- or FITC-conjugated secondary antibodies (1:1,000, Jackson ImmunoResearch), then washed in PBS. The stained sections were examined with a Leica fluorescence microscope, and images were captured with a CCD Spot camera.

### Data analysis

Data are expressed as the mean ± SEM. Behavioral data were analyzed by two-way ANOVA (Time × Treatment) followed by Bonferroni post hoc test. For the analysis of GFAP or IBA-1 immunoreactivity, the images of the spinal cord dorsal horn were captured, the laminae I-IV of the spinal cord section (6 sections for each animal) were outlined, and a numerical value of the intensity was calculated with a computer-assisted imaging analysis system (Image J). The intensity of the background was subtracted in each section and the GFAP or IBA-1 intensity was expressed as fold increase compared to control. Immunostaining and RT-PCR data were analyzed by one-way ANOVA followed by Bonferroni post hoc test. Differences with P < 0.05 were considered statistically significant.

## Author Contributions

J.J.C. and L.D. carried out the animal model, behavioral testing, RT-PCR, and immunohistochemistry experiments. JJC also analyzed the data and drafted the manuscript. L.X.Z. carried out the cell culture experiments and did RT-PCR for cell lysates. X.Z. participated to behavioral tests. S.C. participated to the design of the experiments. Y.J.G. conceived of the project, coordinated and supervised the experiments, and revised the manuscript. All authors reviewed the final manuscript.

## Additional Information

**How to cite this article**: Chen, J.-J. *et al*. Intrathecal curcumin attenuates pain hypersensitivity and decreases spinal neuroinflammation in rat model of monoarthritis. *Sci. Rep.* **5**, 10278; doi: 10.1038/srep10278 (2015).

## References

[b1] Maheshwari R. K., Singh A. K., Gaddipati J. & Srimal R. C. Multiple biological activities of curcumin: a short review. Life Sci. 78, 2081–2087 (2006). 16413584 10.1016/j.lfs.2005.12.007

[b2] Strimpakos A. S. & Sharma R. A. Curcumin: preventive and therapeutic properties in laboratory studies and clinical trials. Antioxid Redox Signal 10, 511–545 (2008). 18370854 10.1089/ars.2007.1769

[b3] Mittal N., Joshi R., Hota D. & Chakrabarti A. Evaluation of antihyperalgesic effect of curcumin on formalin-induced orofacial pain in rat. Phytother Res. 23, 507–512 (2009). 19051211 10.1002/ptr.2662

[b4] Tajik H., Tamaddonfard E. & Hamzeh-Gooshchi N. The effect of curcumin (active substance of turmeric) on the acetic acid-induced visceral nociception in rats. Pak. J. Biol. Sci. 11, 312–314 (2008). 18817212 10.3923/pjbs.2008.312.314

[b5] Jeon Y. *et al.* Curcumin could prevent the development of chronic neuropathic pain in rats with peripheral nerve injury. Curr. Ther. Res. Clin. Exp. 74, 1–4 (2013). 24385078 10.1016/j.curtheres.2012.10.001 PMC3862204

[b6] Sharma S., Kulkarni S. K., Agrewala J. N. & Chopra K. Curcumin attenuates thermal hyperalgesia in a diabetic mouse model of neuropathic pain. Eur. J. Pharmacol. 536, 256–261 (2006). 16584726 10.1016/j.ejphar.2006.03.006

[b7] Al Moundhri M. S., Al-Salam S., Al Mahrouqee A., Beegam S. & Ali B. H. The effect of curcumin on oxaliplatin and cisplatin neurotoxicity in rats: some behavioral, biochemical, and histopathological studies. J. Med. Toxicol. 9, 25–33 (2013). 22648527 10.1007/s13181-012-0239-x PMC3576489

[b8] Mun S. H. *et al.* Oral administration of curcumin suppresses production of matrix metalloproteinase (MMP)-1 and MMP-3 to ameliorate collagen-induced arthritis: inhibition of the PKCdelta/JNK/c-Jun pathway. J. Pharmacol. Sci. 111, 13–21 (2009). 19763044 10.1254/jphs.09134fp

[b9] Breivik H., Collett B., Ventafridda V., Cohen R. & Gallacher D. Survey of chronic pain in Europe: prevalence, impact on daily life, and treatment. Eur. J. Pain. 10, 287–333 (2006). 16095934 10.1016/j.ejpain.2005.06.009

[b10] Sandur S. K. *et al.* Curcumin, demethoxycurcumin, bisdemethoxycurcumin, tetrahydrocurcumin and turmerones differentially regulate anti-inflammatory and anti-proliferative responses through a ROS-independent mechanism. Carcinogenesis 28, 1765–1773 (2007). 17522064 10.1093/carcin/bgm123

[b11] Troselj K. G. & Kujundzic R. N. Curcumin in combined cancer therapy. Curr. Pharm. Des. 20, 6682–6696 (2014). 25341940 10.2174/1381612820666140826154601

[b12] Zhang Z. J. *et al.* Curcumin inhibits LPS-induced CCL2 expression via JNK pathway in C6 rat astrocytoma cells. Cell Mol. Neurobiol. 32, 1003–1010 (2012). 22410671 10.1007/s10571-012-9816-4 PMC11498555

[b13] Abe Y., Hashimoto S. & Horie T. Curcumin inhibition of inflammatory cytokine production by human peripheral blood monocytes and alveolar macrophages. Pharmacol. Res. 39, 41–47 (1999). 10051376 10.1006/phrs.1998.0404

[b14] Chen Y. R. & Tan T. H. Inhibition of the c-Jun N-terminal kinase (JNK) signaling pathway by curcumin. Oncogene 17, 173–178 (1998). 9674701 10.1038/sj.onc.1201941

[b15] Lee K. W., Kim J. H., Lee H. J. & Surh Y. J. Curcumin inhibits phorbol ester-induced up-regulation of cyclooxygenase-2 and matrix metalloproteinase-9 by blocking ERK1/2 phosphorylation and NF-kappaB transcriptional activity in MCF10A human breast epithelial cells. Antioxid Redox Signal 7, 1612–1620 (2005). 16356124 10.1089/ars.2005.7.1612

[b16] Zhong Y., Liu T. & Guo Z. Curcumin inhibits ox-LDL-induced MCP-1 expression by suppressing the p38MAPK and NF-kappaB pathways in rat vascular smooth muscle cells. Inflamm. Res. 61, 61–67 (2011). 22005927 10.1007/s00011-011-0389-3

[b17] Ji R. R., Gereau R. W. t., Malcangio M. & Strichartz G. R. MAP kinase and pain. Brain Res Rev 60, 135–148 (2009). 19150373 10.1016/j.brainresrev.2008.12.011 PMC2666786

[b18] Zhao L. X., Jiang B. C., Wu X. B., Cao D. L. & Gao Y. J. Ligustilide attenuates inflammatory pain via inhibition of NFkappaB-mediated chemokines production in spinal astrocytes. Eur. J. Neurosci. 39, 1391–1402 (2014). 24521480 10.1111/ejn.12502

[b19] Gao Y. J. & Ji R. R. Chemokines, neuronal-glial interactions, and central processing of neuropathic pain. Pharmacol. Ther. 126, 56–68 (2010). 20117131 10.1016/j.pharmthera.2010.01.002 PMC2839017

[b20] Kiguchi N., Kobayashi Y. & Kishioka S. Chemokines and cytokines in neuroinflammation leading to neuropathic pain. Curr. Opin. Pharmacol. 12, 55–61 (2012). 22019566 10.1016/j.coph.2011.10.007

[b21] White F. A., Jung H. & Miller R. J. Chemokines and the pathophysiology of neuropathic pain. Proc. Natl. Acad. Sci. USA 104, 20151–20158 (2007). 18083844 10.1073/pnas.0709250104 PMC2154400

[b22] DeLeo J. A. & Yezierski R. P. The role of neuroinflammation and neuroimmune activation in persistent pain. Pain 90, 1–6 (2001). 11166964 10.1016/s0304-3959(00)00490-5

[b23] Milligan E. D. & Watkins L. R. Pathological and protective roles of glia in chronic pain. Nat. Rev. Neurosci. 10, 23–36 (2009). 19096368 10.1038/nrn2533 PMC2752436

[b24] Ji R. R., Xu Z. Z., Strichartz G. & Serhan C. N. Emerging roles of resolvins in the resolution of inflammation and pain. Trends Neurosci. 34, 599–609 (2011). 21963090 10.1016/j.tins.2011.08.005 PMC3200462

[b25] Abbadie C. *et al.* Chemokines and pain mechanisms. Brain Res. Rev. 60, 125–134 (2009). 19146875 10.1016/j.brainresrev.2008.12.002 PMC2691997

[b26] Gao Y. J. *et al.* JNK-induced MCP-1 production in spinal cord astrocytes contributes to central sensitization and neuropathic pain. J. Neurosci. 29, 4096–4108 (2009). 19339605 10.1523/JNEUROSCI.3623-08.2009 PMC2682921

[b27] Zhang R. X. *et al.* Interleukin 1beta facilitates bone cancer pain in rats by enhancing NMDA receptor NR-1 subunit phosphorylation. Neuroscience 154, 1533–1538 (2008). 18554806 10.1016/j.neuroscience.2008.04.072 PMC2495055

[b28] Kawasaki Y., Zhang L., Cheng J. K. & Ji R. R. Cytokine mechanisms of central sensitization: distinct and overlapping role of interleukin-1beta, interleukin-6, and tumor necrosis factor-alpha in regulating synaptic and neuronal activity in the superficial spinal cord. J. Neurosci. 28, 5189–5194 (2008). 18480275 10.1523/JNEUROSCI.3338-07.2008 PMC2408767

[b29] Colpaert F. C. Evidence that adjuvant arthritis in the rat is associated with chronic pain. Pain 28, 201–222 (1987). 3547255 10.1016/0304-3959(87)90117-5

[b30] Schaible H. G., Ebersberger A. & Von Banchet G. S. Mechanisms of pain in arthritis. Ann. N Y Acad. Sci. 966, 343–354 (2002). 12114291 10.1111/j.1749-6632.2002.tb04234.x

[b31] Shan S. *et al.* Is functional state of spinal microglia involved in the anti-allodynic and anti-hyperalgesic effects of electroacupuncture in rat model of monoarthritis? Neurobiol. Dis. 26, 558–568 (2007). 17442579 10.1016/j.nbd.2007.02.007 PMC2681292

[b32] Romero-Sandoval E. A., Horvath R. J. & DeLeo J. A. Neuroimmune interactions and pain: focus on glial-modulating targets. Curr. Opin. Investig Drugs 9, 726–734 (2008). PMC2696046 18600578

[b33] Marchand F., Perretti M. & McMahon S. B. Role of the immune system in chronic pain. Nat. Rev. Neurosci. 6, 521–532 (2005). 15995723 10.1038/nrn1700

[b34] Julius D. & Basbaum A. I. Molecular mechanisms of nociception. Nature 413, 203–210 (2001). 11557989 10.1038/35093019

[b35] Watkins L. R., Milligan E. D. & Maier S. F. Glial proinflammatory cytokines mediate exaggerated pain states: implications for clinical pain. Adv. Exp. Med. Biol. 521, 1–21 (2003). 12617561

[b36] Gao Y. J. & Ji R. R. Targeting astrocyte signaling for chronic pain. Neurotherapeutics 7, 482–493 (2010). 20880510 10.1016/j.nurt.2010.05.016 PMC2950097

[b37] Yeon K. Y. *et al.* Curcumin produces an antihyperalgesic effect via antagonism of TRPV1. J. Dent. Res. 89, 170–174 (2010). 20040737 10.1177/0022034509356169

[b38] Zhao X. *et al.* Curcumin exerts antinociceptive effects in a mouse model of neuropathic pain: descending monoamine system and opioid receptors are differentially involved. Neuropharmacology 62, 843–854 (2012). 21945716 10.1016/j.neuropharm.2011.08.050

[b39] Zhu X. *et al.* Curcumin alleviates neuropathic pain by inhibiting p300/CBP histone acetyltransferase activity-regulated expression of BDNF and cox-2 in a rat model. PloS one 9, e91303 (2014). 24603592 10.1371/journal.pone.0091303 PMC3946321

[b40] Ji F. T., Liang J. J., Liu L., Cao M. H. & Li F. Curcumin exerts antinociceptive effects by inhibiting the activation of astrocytes in spinal dorsal horn and the intracellular extracellular signal-regulated kinase signaling pathway in rat model of chronic constriction injury. Chin Med. J. (Engl.) 126, 1125–1131 (2013). 23506591

[b41] Tsai Y. M., Chien C. F., Lin L. C. & Tsai T. H. Curcumin and its nano-formulation: the kinetics of tissue distribution and blood-brain barrier penetration. Int. J. Pharm. 416, 331–338 (2011). 21729743 10.1016/j.ijpharm.2011.06.030

[b42] Li Y. *et al.* Curcumin attenuates diabetic neuropathic pain by downregulating TNF-alpha in a rat model. Int. J. Med. Sci. 10, 377–381 (2013). 23471081 10.7150/ijms.5224 PMC3590595

[b43] Han Y. K. *et al.* Analgesic effects of intrathecal curcumin in the rat formalin test. Korean J. Pain 25, 1–6 (2012). 22259709 10.3344/kjp.2012.25.1.1 PMC3259131

[b44] Ji R. R., Berta T. & Nedergaard M. Glia and pain: is chronic pain a gliopathy? Pain 154 Suppl 1, S10–28 (2013). 23792284 10.1016/j.pain.2013.06.022 PMC3858488

[b45] Mika J. *et al.* Differential activation of spinal microglial and astroglial cells in a mouse model of peripheral neuropathic pain. Eur. J. Pharmacol. 623, 65–72 (2009). 19766105 10.1016/j.ejphar.2009.09.030

[b46] Raghavendra V., Tanga F. & DeLeo J. A. Inhibition of microglial activation attenuates the development but not existing hypersensitivity in a rat model of neuropathy. J. Pharmacol. Exp. Ther. 306, 624–630 (2003). 12734393 10.1124/jpet.103.052407

[b47] Gao Y. J. & Ji R. R. Light touch induces ERK activation in superficial dorsal horn neurons after inflammation: involvement of spinal astrocytes and JNK signaling in touch-evoked central sensitization and mechanical allodynia. J Neurochem. 115, 505–514 (2010). 20722971 10.1111/j.1471-4159.2010.06946.x PMC2970698

[b48] Sun S. *et al.* New evidence for the involvement of spinal fractalkine receptor in pain facilitation and spinal glial activation in rat model of monoarthritis. Pain 129, 64–75 (2007). 17123734 10.1016/j.pain.2006.09.035

[b49] Lim G. P. *et al.* The curry spice curcumin reduces oxidative damage and amyloid pathology in an Alzheimer transgenic mouse. J. Neurosci. 21, 8370–8377 (2001). 11606625 10.1523/JNEUROSCI.21-21-08370.2001 PMC6762797

[b50] Zhu H. T. *et al.* Curcumin attenuates acute inflammatory injury by inhibiting the TLR4/MyD88/NF-kappaB signaling pathway in experimental traumatic brain injury. *J. Neuroinflammation* 11, 59 (2014). 24669820 10.1186/1742-2094-11-59 PMC3986937

[b51] Hanazawa S. *et al.* Tumor necrosis factor-alpha induces expression of monocyte chemoattractant JE via fos and jun genes in clonal osteoblastic MC3T3-E1 cells. J. Biol. Chem. 268, 9526–9532 (1993). 8486642

[b52] Berta T. *et al.* Extracellular caspase-6 drives murine inflammatory pain via microglial TNF-alpha secretion. J. Clin. Invest. 124, 1173–1186 (2014). 24531553 10.1172/JCI72230 PMC3934175

[b53] Zhang R. X. *et al.* Spinal glial activation in a new rat model of bone cancer pain produced by prostate cancer cell inoculation of the tibia. Pain 118, 125–136 (2005). 16154703 10.1016/j.pain.2005.08.001

[b54] Matsushita K. *et al.* Chemokine (C-C motif) receptor 5 is an important pathological regulator in the development and maintenance of neuropathic pain. Anesthesiology 120, 1491–1503 (2014). 24589480 10.1097/ALN.0000000000000190

[b55] Milligan E. D. *et al.* Spinal glia and proinflammatory cytokines mediate mirror-image neuropathic pain in rats. J. Neurosci. 23, 1026–1040 (2003). 12574433 10.1523/JNEUROSCI.23-03-01026.2003 PMC6741915

[b56] Wei F., Guo W., Zou S., Ren K. & Dubner R. Supraspinal glial-neuronal interactions contribute to descending pain facilitation. J. Neurosci. 28, 10482–10495 (2008). 18923025 10.1523/JNEUROSCI.3593-08.2008 PMC2660868

[b57] Lu Y. *et al.* TRAF6 upregulation in spinal astrocytes maintains neuropathic pain by integrating TNF-alpha and IL-1beta signaling. Pain 155, 2618–2629 (2014). 25267210 10.1016/j.pain.2014.09.027 PMC4250420

[b58] Singh A. K. & Vinayak M. Curcumin Attenuates CFA Induced Thermal Hyperalgesia by Modulation of Antioxidant Enzymes and Down Regulation of TNF-alpha, IL-1beta and IL-6. Neurochem. Res. 40, 463–472 (2014). 25479948 10.1007/s11064-014-1489-6

[b59] Hylden J. L. & Wilcox G. L. Intrathecal morphine in mice: a new technique. Eur. J. Pharmacol. 67, 313–316 (1980). 6893963 10.1016/0014-2999(80)90515-4

[b60] Dixon W. J. Staircase bioassay: the up-and-down method. Neurosci. Biobehav Rev. 15, 47–50 (1991). 2052197 10.1016/s0149-7634(05)80090-9

[b61] Hui J., Zhang Z. J., Zhang X., Shen Y. & Gao Y. J. Repetitive hyperbaric oxygen treatment attenuates complete Freund’s adjuvant-induced pain and reduces glia-mediated neuroinflammation in the spinal cord. J. Pain 14, 747–758 (2013). 23680474 10.1016/j.jpain.2013.02.003

